# Relative Prevalence of Grapevine Leafroll-Associated Virus Species in Wine Grape-Growing Regions of California

**DOI:** 10.1371/journal.pone.0142120

**Published:** 2015-11-03

**Authors:** Abhineet M. Sharma, Breanna Baraff, John T. Hutchins, Michelle K. Wong, G. Kai Blaisdell, Monica L. Cooper, Kent M. Daane, Rodrigo P. P. Almeida

**Affiliations:** 1 Department of Environmental Science, Policy and Management, University of California, Berkeley, California, 94720, United States of America; 2 University of California Cooperative Extension, 1710 Soscol Avenue, Suite 4, Napa, CA, 94559, United States of America; UC Davis MIND Institute, UNITED STATES

## Abstract

Some diseases manifest as one characteristic set of symptoms to the host, but can be caused by multiple pathogens. Control treatments based on plant symptoms can make it difficult to effectively manage such diseases, as the biology of the underlying pathogens can vary. Grapevine leafroll disease affects grapes worldwide, and is associated with several viral species in the family *Closteroviridae*. Whereas some of the viruses associated with this disease are transmitted by insect vectors, others are only graft-transmissible. In three regions of California, we surveyed vineyards containing diseased vines and screened symptomatic plants for all known viral species associated with grapevine leafroll disease. Relative incidence of each virus species differed among the three regions regions, particularly in relation to species with known vectors compared with those only known to be graft-transmitted. In one region, the pathogen population was dominated by species not known to have an insect vector. In contrast, populations in the other surveyed regions were dominated by virus species that are vector-transmissible. Our survey did not detect viruses associated with grapevine leafroll disease at some sites with characteristic disease symptoms. This could be explained either by undescribed genetic diversity among these viruses that prevented detection with available molecular tools at the time the survey was performed, or a misidentification of visual symptoms that may have had other underlying causes. Based on the differences in relative prevalence of each virus species among regions and among vineyards within regions, we expect that region and site-specific management strategies are needed for effective disease control.

## Introduction

The control and management of diseases is predicated on the accurate identification of their etiology. In plant pathology, many diseases are caused by individual pathogens, allowing scientists and stakeholders to devise and implement targeted strategies aimed at reducing disease incidence, as well as limiting economic, environmental, and social impacts that may occur as a consequence of the diseased plants. However, there are also examples of diseases caused by pathogen complexes; therefore, in some cases a set of multiple factors is required for disease symptom expression, while in other examples infection by one member of a group of related pathogens may lead to similar disease symptoms [1,2]. In the latter scenario, site- or region-specific identification of pathogens associated with disease symptoms is important, as each pathogen may have distinct biological traits and therefore requires a different strategy to limit its impact and spread.

Grapevine leafroll disease (GLD) is caused by a complex of several ssRNA virus species in the family *Closteroviridae*; this family has four genera, three of which contain viruses associated with GLD [3]. *Grapevine leafroll-associated virus 2* (GLRaV-2) is in the genus *Closterovirus*, which includes aphid-transmitted viruses and other species that lack a known vector, such as the graft-transmissible GLRaV-2 [3]. Different variants of GLRaV-2 cause distinct GLD symptoms, yet there has been no evidence of a vector and the pattern of infected vines indicate that GLRaV-2 is disseminated only through the planting of virus-infected plant material [4]. On the other hand, viruses in the genus *Ampelovirus*, including *Grapevine leafroll-associated virus 1* (GLRaV-1), *Grapevine leafroll-associated virus 3* (GLRaV-3), and the *Grapevine leafroll-associated virus 4* group (herein GLRaV-4LV) are transmitted primarily by mealybug vectors [5–10]. There are several genetically distinct variants of GLRaV-3, which can affect each other after mealybug-borne introduction to an uninfected host [11]. It should be noted that recent studies have collapsed several virus species associated with GLRaV-4 into GLRaV-4LV (LV standing for ‘like viruses’), due to the lack of substantial genetic differences among those species [3]. Accordingly, these viruses are associated with GLD and can be spread by insect vectors as well as contaminated plant material. Finally, *Grapevine leafroll-associated virus 7* (GLRaV-7) is a member of the newly proposed genus *Velarivirus*. There are no vectors identified for GLRaV-7, and its role in GLD has been recently questioned [12,13]. Thus, GLD is associated with several viral species that have broad biological differences, including varying associations with disease symptoms and insect vectors. Importantly, current knowledge regarding the biological role of these viruses in GLD is primarily derived from correlational evidence based on surveys and experimental inoculations by grafting infected wood or manipulating insect vectors.

GLD has reemerged as a disease of economic importance during the past few decades, primarily because its incidence was shown to increase in most grape-growing regions of the world in the presence of mealybug vectors [2]. However, because the etiology of GLD is complex, management strategies must also consider the virus species primarily associated with local and regional epidemics. This factor becomes especially relevant because grapevine management, including the establishment of new vineyards, is heavily dictated by the need to use specific varieties and clones due to fruit qualitative traits derived from such choices. Grapevines are perennial plants and because the competitive wine market is based on wine quality, stakeholders selecting plant material have historically prioritized fruit characteristics over certified plant material. Educational efforts detailing the detrimental effect of grapevine viruses and the benefits of certified material, combined with advances in pathogen detection technology have increasingly led to the selection of certified planting material. Consequently, GLD management approaches must vary according to the viruses associated with plants.

California has a few major wine growing regions, along with a handful of regions with smaller production. Major growing regions include the North Coast, Central Coast, and Central Valley, the Sierra Foothills represents a smaller region [14]. GLD has been studied extensively in the North Coast, especially Napa Valley [15–18], where GLRaV-3 is the primary virus species associated with this disease [15,16]. GLRaV-3 has been found in multiple cultivars of *Vitis vinifera*, as well as the native grape species, *V*. *californica* [15,17]. Furthermore, seasonal variation in within-host virus populations has been observed [18]. Considerably less information exists regarding GLD in other regions of California.

In this study, we surveyed three grape-growing regions of California for the presence of GLD-associated viruses. Our results show that relative prevalence of the various GLRaV species differed among wine growing regions within California, and that substantial variability in virus presence occurs among vineyards within regions. Ultimately, our results indicate that management of GLD requires knowledge about the range of viruses associated with afflicted vineyards, rather than relying solely on the visual identification of disease symptoms.

## Materials and Methods

### Sampling structure

A total of twenty-five vineyard blocks in three wine growing regions were surveyed, which included *Vitis vinifera* cvs. Barbera, Cabernet franc, Cabernet Sauvignon, Gamay, Merlot, Petite Syrah, Pinot noir, Syrah, and Zinfandel (available information about all sites is provided in S1 File). Only red-berried wine grape cultivars were selected because white-berried varieties are difficult to accurately diagnose visually [3]. All vines sampled had visual symptoms characteristic of GLD, including leaf reddening and downward rolling [2,3]. We sampled five sites in San Luis Obispo County (Edna Valley and Paso Robles appellations), located in the Central Coast region. In the Central Valley we sampled nine sites in San Joaquin County (Lodi, Mokelumne River, and Jahant appellations). In the Sierra Foothills region, located east of the Central Valley region, we sampled eleven sites in Amador and El Dorado Counties (California Shenandoah Valley, Fiddletown, and El Dorado appellations) [14]. All samples were collected (by at least one experienced individual for each site) in August, September, and October 2010, during which characteristic GLD symptoms were easily identifiable and viral populations would be high enough for reliable detection [18]. The blocks were selected based on grower participation, the presence of characteristic foliar GLD symptoms, and information indicating recent pathogen spread (increased disease incidence) if available. Information on pathogen spread was anecdotal and not confirmed by multiple years of field surveys. We excluded vineyard blocks that had been knowingly established with virus-infected plant material. In order to protect the anonymity of participating growers, a detailed geographic location of each site cannot be made publically available. The study was carried out on private land with owner permission (no specific permits were required); the field survey did not involve endangered or protected species. To maximize our likelihood of detection in the event that virus populations may be unevenly distributed within plants, we collected and pooled three petioles from each symptomatic plant for molecular virus testing. A total of 571 plants were tested, with 19–30 vines tested per site: 234 from the Sierra Foothills, 149 from the Central Coast, and 188 from the Central Valley.

### Molecular testing for GLRaV infection

All petiole samples were stored at -80°C until RNA extractions were performed from 100 mg of pooled petiole samples using a modified version of the method from a previous survey of Napa Valley in the North Coast Region [15], based on the protocol used by Osman and others [19]. Each sample was cut with a razor blade into small pieces and placed into a 2.0 ml microcentrifuge tube containing a sterilized .3175 cm chrome ball bearing (Boca Bearings, Delray Beach, FL) and 1.8 ml of extraction buffer (1.59 g/l Na_2_CO_3_, 2.93 g/l NaHCO_3_, pH 9.6 containing 2% PVP-40, 0.2% bovine serum albumin, 0.05% Tween 20 and 1% Na_2_S2O_5_). Samples were macerated using a Precellys 24 Tissue homogenizer (Bertin Technologies, Catalog 03119.200.RD000) run at 6,500 Hz for two 10 sec cycles with a 30 sec intermission between cycles. Following maceration, samples were centrifuged for 10 min at 16,000 rpm and 1.5 ml of supernatant was stored in a new microcentrifuge tube at -20°C. For positive controls for all GLRaV species tested in this study, known infected vine cuttings were provided by the Foundation Plant Services at the University of California, Davis (accessions LR101, LR102, LR106, and LR109) and propagated in the Oxford Tract greenhouse facility at the University of California, Berkeley.

All samples were tested for the presence of GLRaV 1–5, 7 and 9 using a multiplex RT-PCR approach modified from Sharma et al. [15]. The major modifications include the addition of a newly designed GLRaV-7 primer pair and the incorporation of the GLRaV-3 coat protein primer (CP) into the species-level testing [15] (S2 File). The CP primer pair was added to the species level testing in this study due to the inability of the original GLRaV-3 HSP70h primers to detect certain variants. The GLRaV-7 primers were designed for this study based on a consensus of all available coat protein gene sequences at the time of the study (EF093187, EU334662, GQ849392, GQ849393, Y15987) and tested using known GLRaV-7 positive vines. These two primer pairs were used in a separate multiplex reaction to insure that they would not alter our original species level detection methodology. All primer pairs, their appropriate annealing temperatures, and concentrations are listed in Sharma et al. [15] and S2 File. All other steps from Sharma et al. [15] were followed and the plant 18S rRNA was used as an internal control for RNA quality in a fourth, separate reaction. Of note, whereas three different primer sets were used for GLRaV-4, 5 and 9, these were later grouped together as GLRaV-4LV for analysis in order to adhere to current nomenclature standards.

### Sequencing

Following species level detection; 10% of samples positive for each GLRaV species were randomly selected for sequencing. The same method as above was used to prepare the extractions for PCR. The same primers were used for PCR and sequencing for all GLRaV species, except for GLRaV-3. In order to sequence the same 428 bp region of the GLRaV-3 CP gene from previous studies, the primer pairs from our initial Napa study were used for PCR and a second nested primer pair was used for sequencing [15]. For all samples, three independent reactions per sample per strand were run with a final concentration of 500 nM per primer pair and the same thermocycler conditions listed above. After PCR, purification and sequencing were performed at Qintarabio Inc. in Albany, CA. Sequences were thoroughly examined for quality and only those showing a single peak per nucleotide were used. This was done to avoid sequencing mixed infections, which would result in erroneous data. Only samples that provided all six reads were used. Sequences were assembled into consensus sequences using Vector NTI version 11 (Invitrogen, Carlsbad, CA) by overlapping the three independent reads per strand. Sequences were deposited into GenBank (KT213466-KT213549).

### Statistical analyses

To compare GLRaV prevalence among the three regions, a proportion of GLRaV-positive vines were compared among the three regions surveyed, using a generalized linear mixed model with a binomial distribution. Site was specified as a random variable nested within each region. A single-step post hoc test was used to discern statistically significant differences among regions. The same procedure was followed to compare the prevalence of mixed infections among the three regions. In a previous study a similar survey was performed for the North Coast area of Napa Valley [15]; due to methodological similarities and the possibility of generating a more complete overview of GLRaV prevalence in California, analyses were also performed with the addition of that data set. Because samples in the North Coast were not tested for GLRaV-7 [15], the same two comparisons were repeated among all four regions after removing GLRaV-7 from the data (three regions surveyed in this study and the North Coast region previously surveyed).

To compare all GLRaV species among the three regions, the relative proportion of each GLRaV species at each site was calculated by dividing the number of positive samples of each GLRaV species by the total number of positive samples, and each site was used as one replicate in the analysis. Relative abundance of each virus was compared individually among regions using a generalized linear model with a Gaussian distribution. Relative abundance data were arcsine-transformed prior to analysis to better meet the assumptions of the model. We report statistics that are not corrected for multiple tests, but we note that the statistical significance (*P < 0*.*05*) of our results would remain consistent after a Bonferroni-Holm correction for multiple tests [20]. All analyses were performed using R version 3.2.0.

## Results

### Regions differ in occurrence of GLRaVs

**Fig 1:**
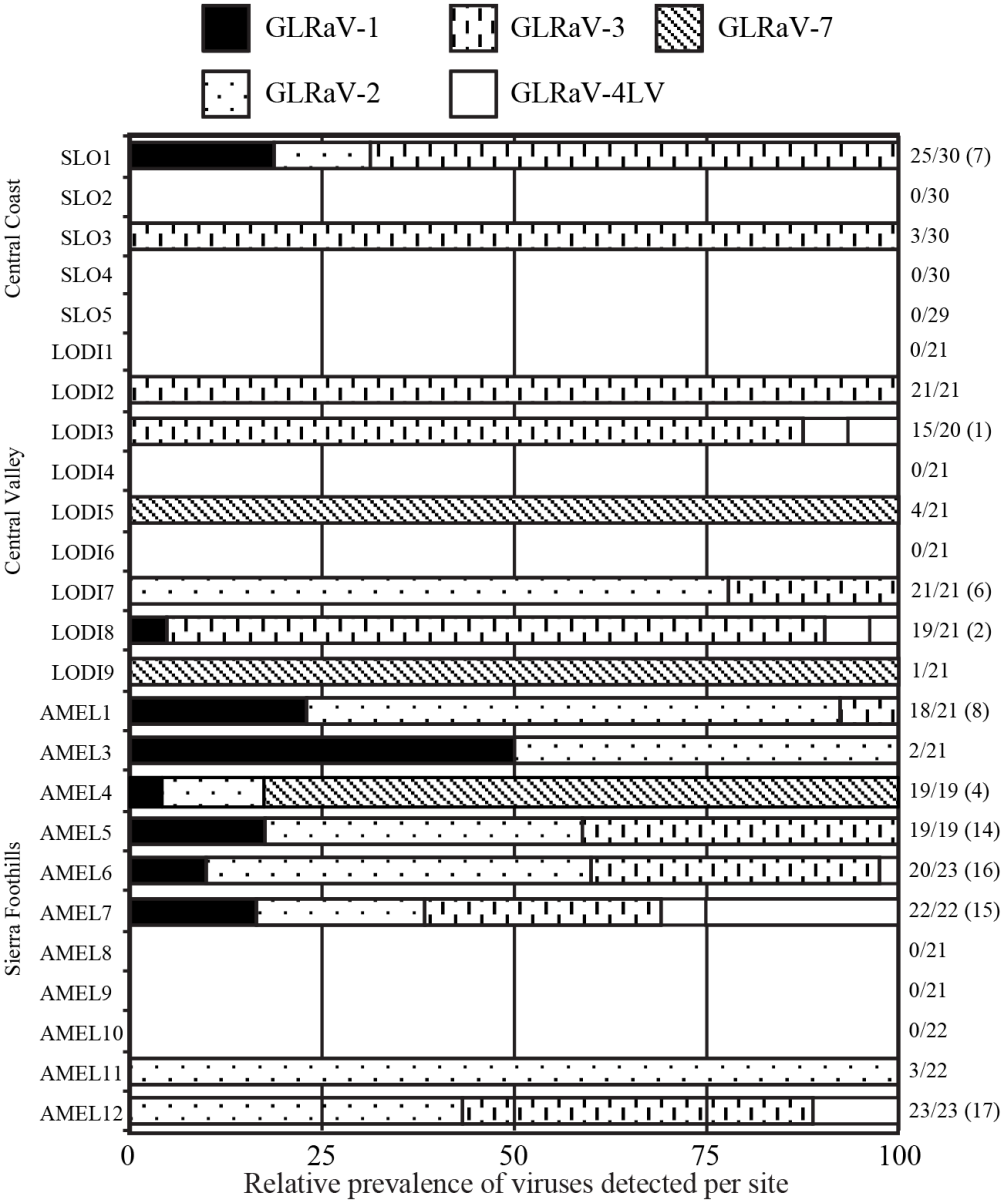
Relative prevalence of grapevine leafroll-associated viruses in tested vineyards. Data for individual vineyards in each of three regions are shown, with the number of positive plants and the total number of plants tested indicated on the right side of each column. Numbers in parentheses indicate the number of plants with mixed infections, if any. Acronyms; SLO, San Luis Obispo County; LODI, San Joaquin County; AMEL, Amador and El Dorado Counties.

When considering overall detection of GLRaV -1, -2, -3, -4LV, and -7, there were significantly fewer samples that tested positive in the Central Coast region than in the other two regions, which did not differ from each other (*z = 2*.*414*, *P = 0*.*0158*, *df = 566*) (Fig 1). When GLRaV-7 was excluded from the analysis and the North Coast was included, we found the highest overall detection of GLRaVs in the Sierra Foothills and North Central Valley (*z = 5*.*505*, *P < 0*.*0001*, *df = 1033)*, followed by the North Coast, and the lowest prevalence was again in the Central Coast.

Prevalence of mixed infections with all GLRaVs included in the analysis was highest in the Sierra Foothills, while the Central Coast and North Central Valley did not differ significantly from each other (*z = 3*.*113*, *P = 0*.*00185*, *df = 566*). When mixed infections were compared among four growing regions with GLRaV-7 excluded, significant differences among regions were found, with mixed infections again being more common in the Sierra Foothills than in the other three regions (*z = 5*.*963*, *P < 0*.*0001*, *df = 1033*).

### Regions differ in relative abundance of GLRaV species

**Fig 2:**
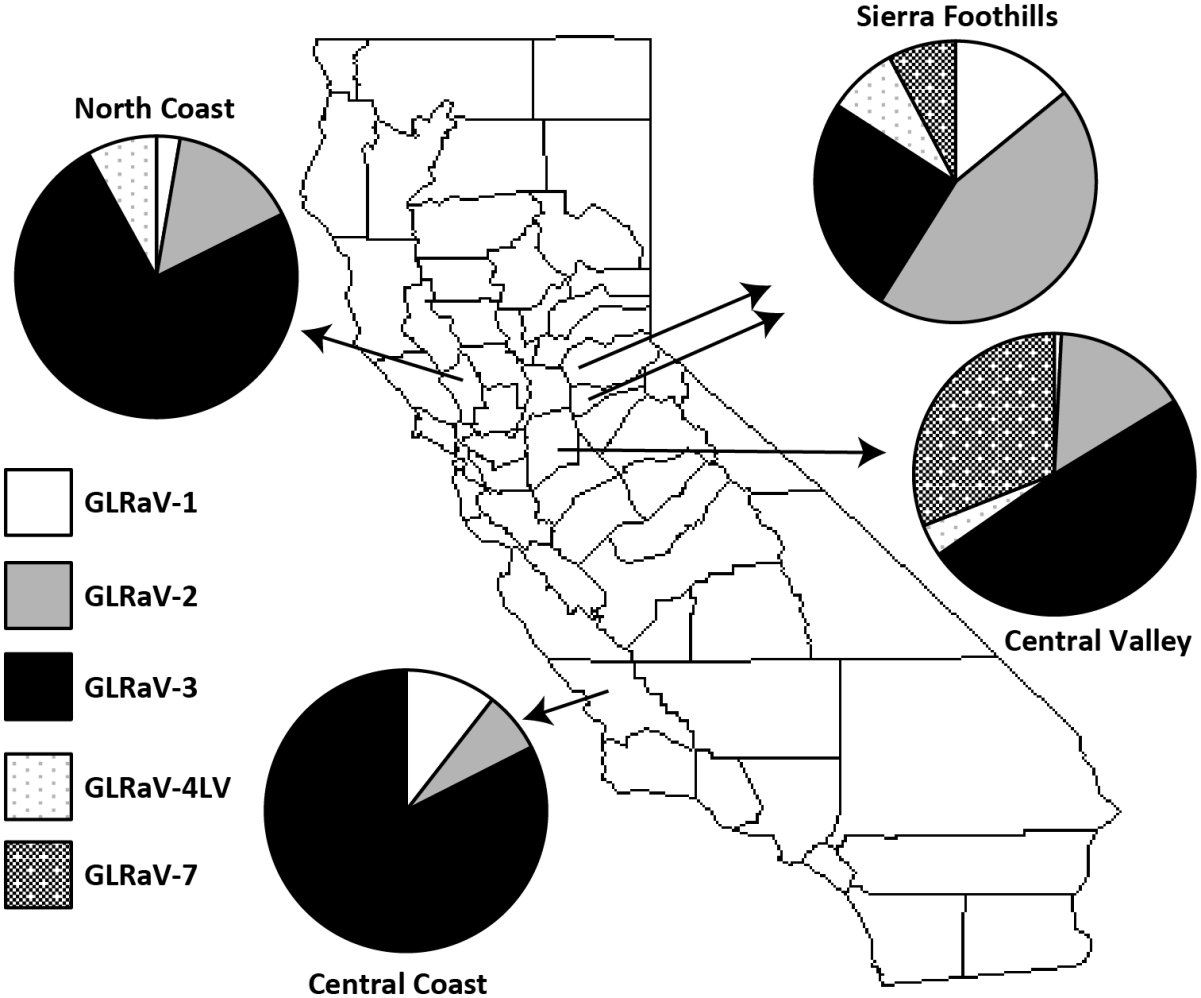
Relative prevalence of grapevine leafroll-associated virus species by region. Data for the North Coast region were obtained from Sharma et al. 2011; GLRaV-7 was not tested in that survey. The map was generated by authors with R version 3.2.0.

Relative abundance of individual GLRaVs varied among regions (Fig 2). GLRaV-1 was highest in relative abundance in the Sierra Foothills, and did not significantly differ between the Central Coast and Central Valley (*F = 22*.*09*, *P = 0*.*0004*, *df = 13*). GLRaV-2 also had the highest relative abundance in the Sierra Foothills, and did not significantly differ between the Central Valley and Central Coast (*F = 37*.*28*, *P < 0*.*0001*, *df = 13*). GLRaV-3 had the highest relative abundance in the Central Coast, while its relative abundance did not significantly differ between the Central Valley and Sierra Foothills (*F = 7*.*68*, *P = 0*.*0152*, *df = 13*). Neither GLRaV-4LV nor GLRaV-7 were found in the Central Coast, and relative abundance of neither GLRaV-4LV (*F = 4*.*28*, *P = 0*.*0589*, *df = 13*) nor GLRaV-7 (*F = 0*.*879*, *P = 0*.*667*, *df = 13*) differed significantly between the Central Valley and Sierra Foothills.

When considering four instead of three regions but excluding GLRaV-7, our measures of the relative abundance of GLRaVs 1–4 were affected slightly in the Central Valley and Sierra Foothills. GLRaV-1 had the highest relative abundance in the Sierra Foothills, intermediate in the Central Coast, and lowest relative abundance in the North Coast and Central Valley (*F = 36*.*16*, *P < 0*.*0001*, *df = 38*). The relative abundance of GLRaV-2 was highest in the Sierra Foothills, intermediate in the Central Valley and North Coast, and lowest in the Central Coast (*F = 47*.*03*, *P < 0*.*0001*, *df = 38*). GLRaV-3 had the highest relative abundance in the Central Coast and North Coast, followed by the Central Valley, with lower relative abundance in the Sierra Foothills (*F = 21*.*71*, *P < 0*.*0001*, *df = 38*). GLRaV-4LV was not found in the Central Coast, and relative abundance of GLRaV-4LV did not vary significantly among the remaining three growing regions (*F = 3*.*07*, *P = 0*.*0875*, *df = 38*).

A total of five sites were surveyed in the Central Coast, two of which were positive for at least one GLRaV species in our detection panel. Approximately 19% of all tested samples were positive for either GLRaV-1, 2, or 3. Only these three viruses were detected in the Central Coast and were found in 4.0%, 2.6%, and 14.8% of all tested samples respectively (n = 149 vines). Within the GLRaV-3 group, there was 100% concordance between the CP and HSP70h primer pairs at one site, indicating that all infections may have consisted of one genetically distinct variant [3]. The other GLRaV-3 positive site in the Central Coast, SLO-3, had three positive samples with the CP primer but none with the HSP70h primer, suggesting infection with a variant that differed from the former site, which may not have been detectable prior to our development of the CP primer pair [15,21]. Of all positive samples, 4.7% were mixed with two or more GLRaV species. We did not detect GLRaV-4LV or GLRaV-7 in the Central Coast region.

A total 188 samples from nine sites were tested in the Central Valley region (San Joaquin County), with planting dates ranging from 1978 to 2007. Of the sites tested, six were positive for one of the viruses in our panel. Of the samples tested, 40% were positive for at least one virus in our panel, of which roughly 53% of all tested samples were positive for GLRaV-3, the most common virus isolated in the region. GLRaV-1, 2, 4LV, and 7 were also detected in the Central Valley (0.9%, 17%, 4.0% and 33% of GLRaV positive samples, respectively). In one surveyed Cabernet Sauvignon block, planted in 1990, every vine tested was positive for GLRaV-3 when tested with the HSP70h primers but only six of the samples were positive when tested with the CP primer set (n = 21), indicating infections with multiple genetically distinct variants of GLRaV-3 within that block. Additionally, vine mealybug (*Planococcus ficus*), a known efficient vector of GLRaV-3 was present at the site [7,22–24]. Two other sites with a high incidence of GLRaV-3-infected vines were adjacent to a vineyard block with both vine mealybug and GLRaV-3. GLRaV-2 was found at just one site, the oldest block (Zinfandel, planted in 1978), where every vine tested positive for GLRaV-2. Some vines at this block also tested positive for GLRaV-3. We found two sites with vines positive for GLRaV-7 but no other viruses. Similar to the Central Coast, mixed infections were detected in 5% of all samples tested.

Eight out of the 11 sites tested in the Sierra Foothills were positive for one of the viruses in our detection panel. A total of 234 samples were tested, of which 47% were positive for at least one virus. In contrast to the other sites, 72% of positive samples were GLRaV-2, the most frequently detected virus in this region. Every positive site had GLRaV-2. GLRaV-3 was the second most common at 40% of positive samples, but was found in only 5 of the 11 tested blocks. GLRaV-1, 4LV, and 7 were also detected in this region (23%, 13%, and 13% of positive samples, respectively). Every sample in AMEL-4, a site planted with non-certified material, was positive for GLRaV-7, the only such block in our study. Of all samples tested, 30% of all samples were mixed infections, notably higher than the other regions.

### Sequencing data

Sequencing data helped elucidate the reason that the CP primer pair was unable to pick up certain variants while the HSP70h primer was positive. The samples that were positive for the HSP70h primer set but negative for the CP primer grouped closely with the “GLRaV-3g” clade from Sharma et al. [15]. At that time, we had identified this as a group that the CP primer pair set failed to detect. Otherwise, our sequencing results did not reveal any novel isolates for any of the sequenced viruses, as all sequences showed high similarity to existing available sequences (E value = 0). All of our sequence data (n = 83) have been deposited to the NCBI database (KT213466-KT213549).

## Discussion

The results of this study highlight the complexity of GLD and the difficulty in implementing disease management practices that are applicable to a broad geographic range of affected grape-growing regions. The within- and between- region variability in virus prevalence, both in regards to the proportion of plants infected, the number of mixed infections, and the viral species present, indicate that control measures should be implemented after diagnostic assays have identified the virus species present at each site. However, broader regional trends must be confirmed with a larger study, so that heterogeneity in cultural practices, grapevine genotype, presence of vectors, and other factors can be incorporated into such an epidemiological survey.

The lack of virus detection in nine of the twenty-five surveyed vineyards is intriguing, as vineyards were selected based on the presence of GLD symptoms. We propose the following possible explanations for this discrepancy. First, we hypothesize that the primer sets used for diagnostics were not capable of amplifying nucleic acids from all virus variants infecting tested plants. PCR primer sets are designed based on available sequence data, and consequently novel or unknown genetically distinct variants of each GLRaV species cannot always be reliably detected. This is highlighted in this study and our previous study [15], as each of two primer pairs that we used differed in their ability to detect genetically distinct variants of GLRaV-3. The recent report of several previously undescribed variants of GLRaV-3 has also highlighted this problem [25]. Second, it is possible that plants were infected with viruses other than GLRaVs, which may cause disease symptoms similar to GLD, such as the newly reported *Grapevine red blotch-associated virus* [26,27]. Furthermore, the less economically important vitiviruses may result in similar disease symptoms [5,17,28]. In New Zealand a recent study showed that visual diagnostics of GLD by trained individuals is efficient in detecting plants infected with GLRaV-3, which is the main virus species in the area surveyed in that study [29]. For scenarios where the composition of GLD-associated viruses in unknown or poorly characterized, such as was the case in this study, we propose that disease symptoms should be used together with molecular and antibody-based detection methods to reduce the rate of potential pathogen misidentification.

With respect to GLRaV incidence, vineyards had two trends within each region. First, some vineyards had no positive plants while others reached 100% GLRaV incidence. Although the discrepancy may be partly due to the limitations of the detection protocols used, as described above. Second, the identity of GLRaV infecting plants varied within and among regions. We speculate that the prevalence of GLD-associated viruses is not only dependent on localized virus spread mediated by vectors, but that a combination of vector spread and establishment of vineyards with virus-infected plant material occurs to varying degrees in all regions. It is also notable that the number of mixed-species infections was higher in the Sierra Foothills region. This trend was largely driven by the high incidence of GLRaV-2, which has no known vector and has not been associated with disease spread in established vineyards [2]. One possible cause is the frequent use of non-certified planting material in the Sierra Foothills region, as reported by vineyard managers.

Although not quantified here, there are substantial differences in the wine grape industry and practices among the surveyed regions. The Sierra Foothills region’s size, climate and relative isolation from other grape-growing regions appear to contribute to lower pest and disease pressure than in the other regions studied. We speculate that this lower pest pressure could result in less frequent removal and replanting of vineyards in that region. The use of certified plant material in these newly planted vineyards may assist in the reduction of GLRaV-2 prevalence. GLRaV-7 also has no known vector and may be similarly reduced by this increased replanting frequency with certified propagation material [2]. As a consequence, the relative prevalence of the vector-transmitted GLRaV-1, GLRaV-3, and GLRaV-4LV would increase in established vineyards; therefore the use of certified material may lead to changes in the viral profile in vineyards. GLRaV-3 is considered the most important GLD-associated virus in most grape-growing regions worldwide [12,22,29–37]. Similarly, GLRaV-3 was the most commonly detected species in this study; however, it is not known why GLRaV-3 is associated with most vector-mediated epidemics of GLD in different countries in the world, while GLRaV-1 and GLRaV-4LV are less problematic [3]. Vector-virus specificity is probably not a major contributor to this pattern, as multiple vector species can transmit multiple GLRaV species [5,6]. A recent study found that GLRaV-3 has higher within-plant viral populations than GLRaV-1 and GLRaV-4LV, and we suggest that this may lead to higher vector-borne transmission efficiency and consequent disease spread [38]. Furthermore, genetically distinct variants of GLRaV-3 are transmitted equally well by the same vector species, but one GLRaV-3 variant can affect the ability of another variant to establish new infections in susceptible hosts [11]. More studies are needed to definitively explain why GLRaV-3 is more prevalent than other vector-borne GLRaVs in California and elsewhere.

Our findings highlight the importance of continued diagnostic testing, including continuing research on grapevine viral diversity, grower education to increase awareness of GLD, and the need to frequently update all stakeholders about advances in current knowledge about GLD and other diseases. Additionally, the regional trends in relative viral prevalence should be taken into account when considering the historical practices in each region and devising future disease management strategies. We acknowledge that grape-growing in California, as well as other regions, is not a uniform agricultural activity and that trends detected in this study should be confirmed with more extensive surveys. Ultimately, a multidisciplinary and collaborative approach that engages researchers, grape growers, and other stakeholders can contribute to effective control of GLD and other economically important diseases in California and worldwide.

## Supporting Information

S1 File GLRaV incidence data by site and region. Worksheet “ThreeRegions” includes data from the regions surveyed for this study for GLRaV-1, -2, -3, -4LV, and -7, as well as varietal and planting date information when available. Worksheet “FourRegions” includes data from the three regions for this study and data from the North Coast region that were collected for Sharma et al. 2011, for GLRaV-1, -2, -3, and -4LV. Worksheet “AbbreviationsExplained” lists the abbreviations that were used in the other two worksheets.
(XLSX)

S2 File Primer sets and multiplex conditions for detection of grapevine leafroll-associated viruses at the species level. Primers were designed by Osman et al. (2007, J. Virol. Methods 141: 22–29). All reactions for virus detection were run in a 3-plex with the first reaction containing the GLRaV-1, GLRaV-2 and GLRaV-3, the second reaction with GLRaV-4, GLRaV-5 and GLRaV-9, a third reaction containing GLRaV-7, CP primer, and a general GLRaV primer, and the fourth reaction with the host plant *18S rRNA* gene alone, which was used as a control. ^1^Primers were designed by Osman et al. (2007, J. Virol. Methods 141: 22–29). ^2^Primers for GLRaV-7 were designed for this study, see methods. ^3^Primers first used in Sharma et. al. 2011 and designed from a consensus of sequences described in Wang et. al. 2010.
(DOCX)
