# Supplementary material for: Relative Prevalence of Grapevine Leafroll-Associated Virus Species in Wine Grape-Growing Regions of California
Source: PLoS One. 2015 Nov 3;10(11):e0142120. doi: 10.1371/journal.pone.0142120 (PMC4631472; doi:10.1371/journal.pone.0142120)
Supplement: S2 File — Primers were designed by Osman et al. (2007, J. Virol. Methods 141: 22–29). All reactions for virus detection were run in a 3-plex with the first reaction containing the GLRaV-1, GLRaV-2 and GLRaV-3, the second reaction with GLRaV-4, GLRaV-5 and GLRaV-9, a third reaction containing GLRaV-7, CP primer, and a general GLRaV primer, and the fourth reaction with the host plant 18S rRNA gene alone, which was used as a control. 1Primers were designed by Osman et al. (2007, J. Virol. Methods 141: 22–29). 2Primers for GLRaV-7 were designed for this study, see methods. 3Primers first used in Sharma et. al. 2011 and designed from a consensus of sequences described in Wang et. al. 2010. (DOCX) [file pone.0142120.s002.docx]

**Supplementary Material S2. Primer sets and multiplex conditions for detection of grapevine leafroll-associated viruses at the species level.**

| GLRaV | Probe sequence (5’ to 3’) | Dye | Actual size (bp) | Plex | Concentration |
| --- | --- | --- | --- | --- | --- |
| 1^1^ | F: ACC TGG TTG AAC GAG ATC GCTT | VIC | 168 | 1 | 400 nM |
|  | R: GTA AAC GGG TGT TCT TCA ATT CTC T |  |  |  |  |
|  |  |  |  |  |  |
| 2^1^ | F: CAT TAT ATT CTT CAT GCC TCT CAG GAT | 6-FAM | 116 | 1 | 400 nM |
|  | R: GAT GAC AAC TTC TGT CCG CTA TAG C |  |  |  |  |
|  |  |  |  |  |  |
| 3^1^ | F: NED-AAG TGC TCT AGT TAA GGT CAG GAG TGA | NED | 254 | 1 | 400 nM |
|  | R: GTA TTG GAC TAC CTT TCG GGA AAA T |  |  |  |  |
| 4^1^ | F: ATA TAC ATA CCA ACC GTT GTG GGT ATA A | 6-FAM | 93 | 2 | 400 nM |
|  | R: CCC TAT AAA CTA GCA CAT CCT TCT CTA GT |  |  |  |  |
|  |  |  |  |  |  |
| 5^1^ | F: AAC ACT CTG CTT TTC TGC TGG C | VIC | 162 | 2 | 400 nM |
|  | R: CTT TTT ATG TCC CGA TAA ACG AGT ACA |  |  |  |  |
|  |  |  |  |  |  |
| 9^1^ | F: CGG CAT AAG AAA AGA TGG CAC | NED | 82 | 2 | 400 nM |
|  | R: TCT TTA TGT CAC GGT AGA CCA ACA C |  |  |  |  |
| 7^2^ | F: TAT ATC CCA ACG GAG ATG GCA ATT TA | PET | 391 | 3 | 400 nM |
|  | R: CCT ATC TCC TTC AAG AGA GAT TTC TG |  |  |  |  |
|  |  |  |  |  |  |
| CP^3^ | F: GAA CTG AAA TTA GGG CAG ATA TA | FAM | 320 | 3 | 400 nM |
|  | R:AAR AAC TTG TCT GGA TCY TT |  |  |  |  |
|  |  |  |  |  |  |
| Gen^1^ | F: TYG GGA CGA CGT TYT CAN C | NED | 490 | 3 | 400 nM |
|  | R: GGY TCR TTC ACI ACI GCY TGI AC |  |  |  |  |
| 18 S^1^ | F: GTG ACG GAG AAT TAG GGT TCG | PET | 70 | 4 | 200 nM |
|  | R: CTG CCT TCC TTG GAT GTG GTA |  |  |  |  |

Primers were designed by Osman et al. (2007, J. Virol. Methods 141: 22-29). All reactions for virus detection were run in a 3-plex with the first reaction containing the GLRaV-1, GLRaV-2 and GLRaV-3, the second reaction with GLRaV-4, GLRaV-5 and GLRaV-9, a third reaction containing GLRaV-7, CP primer, and a general GLRaV primer, and the fourth reaction with the host plant *18S rRNA* gene alone, which was used as a control. ^1^Primers were designed by Osman et al. (2007, J. Virol. Methods 141: 22-29). ^2^Primers for GLRaV-7 were designed for this study, see methods. ^3^Primers first used in Sharma et. al. 2011 and designed from a consensus of sequences described in Wang et. al. 2010.
